# Neutrophil extracellular traps in colorectal cancer: a paradoxical nexus of tumor defense and disease progression

**DOI:** 10.3389/fimmu.2026.1858493

**Published:** 2026-09-18

**Authors:** Yuansha Ge, Weijia Kong, Yuxiao Qian, Yuting Sun, Guanghui Zhu, Jie Li

**Affiliations:** 1 Guang’anmen Hospital, China Academy of Chinese Medical Sciences, Beijing, China; 2 Department of Integrated Chinese and Western Medicine, Zhejiang Cancer Hospital, Hangzhou Institute of Medicine (HIM), Chinese Academy of Sciences, Hangzhou, Zhejiang, China; 3 Graduate School, Beijing University of Chinese Medicine, Beijing, China

**Keywords:** colorectal cancer, NEtosis, NETs formation, neutrophil extracellular traps, tumor microenvironment

## Abstract

Neutrophil extracellular traps (NETs) are extracellular networks of decondensed chromatin and granular proteins generated by neutrophils in response to activating stimuli. Growing evidence indicates that NETs participate in colorectal cancer (CRC) development through their interactions with chronic inflammation, immune regulation, coagulation, and tumor progression. However, their biological effects vary across disease settings, and the regulatory mechanisms specific to CRC have not been fully clarified. This review examines the roles of NETs at different stages of CRC, including the transition from inflammation to cancer, primary tumor growth, metastatic spread, treatment resistance, and CRC-associated complications. We discuss CRC-related mechanisms of NET formation involving tumor-derived chemokines, stromal cells, microbial dysbiosis, oncogenic signals, and ROS-dependent pathways. Available evidence also suggests that NETs are not uniformly pro-tumorigenic. We therefore describe a “paradoxical nexus” in which their effects depend on tumor genotype, neutrophil phenotype, immune context, and the stimulus inducing NET formation. In addition, we review NET-related biomarkers, detection methods, and therapeutic approaches, while considering the evidence required for their clinical application and for context-specific treatment of CRC.

## 1 Introduction

Colorectal cancer (CRC) ranks third in global cancer incidence and second in cancer mortality (1), and its worldwide burden continues to grow. The 2023 Global Burden of Disease study reported 2.29 million new CRC cases and 1.11 million deaths worldwide, marking a 142.7% rise in incidence and a 90.2% rise in mortality compared with the 1990 estimates (2). CRC development is shaped not only by alterations in epithelial cells but also by interactions between tumor cells and the tumor microenvironment (TME). The TME comprises immune and stromal cells, extracellular matrix components, soluble mediators, and blood and lymphatic vessels (3). Myeloid cells are abundant within this environment and can influence tumor growth and progression (4). Neutrophils are innate immune cells that respond rapidly to inflammation and infection, where they contribute to phagocytosis and microbial clearance (5). In tumors, however, their functions are more diverse. Tumor-associated neutrophils (TANs) are present at different stages of cancer development and may exert either tumor-promoting or tumor-restraining effects, depending on their phenotype and local conditions (6). Most available studies nevertheless associate neutrophil infiltration with tumor progression. Of all leukocyte populations assessed in a pan-cancer cohort of over 3,000 tumors across 14 cancer types, tumor-infiltrating neutrophils correlated most strongly with worse clinical outcomes (7).

In 1996, Takei H et al. (8) discovered neutrophil extracellular traps (NETs), revolutionizing the understanding of neutrophil function. NETs formation typically culminates in a lytic form of neutrophil death termed “NETosis” (9). NETosis is a specialized form of reactive oxygen species (ROS)- and protein-arginine deiminase type 4 (PAD4)-dependent programmed cell death that allows neutrophils to eliminate extracellular pathogens through the release of reticular chromatin structures (10). In the context of cancer, this ancient defense mechanism is hijacked and repurposed into a pro-tumorigenic instrument. CRC cells, stromal cells, and therapeutic stressors (e.g., chemotherapy, surgery) secrete copious amounts of cytokines and damage-associated molecular patterns, which persistently activate and recruit neutrophils to the TME, inducing NETosis (10–12). These disseminated “DNA-protein webs,” found within tumor tissue, the circulation, and even distant organs, profoundly influence CRC proliferation, invasion, metastasis, immune evasion, and therapeutic responsiveness through both their physical architecture and bioactive components. NETs have emerged as a critical bridge linking the three major pathological processes of chronic inflammation, coagulation, and cancer, representing a frontier focus in tumor immunology and metabolic research (13). Although NETs are widely recognized for their tumor-promoting activities in CRC, emerging evidence suggests that NETs may also exert protective effects under specific biological contexts. As an essential component of innate immunity, NETs contribute to pathogen clearance and restrict microbial dissemination (9, 13). Although predominantly associated with tumor-promoting effects, NETs and their associated effector proteins may also exert context-dependent anti-tumor activity under particular immune microenvironmental conditions (6, 10–12). These apparently opposing functions indicate that the biological consequences of NETosis cannot be simply categorized as either tumor-promoting or tumor-suppressive, but rather represent a dynamic balance governed by the genetic characteristics of tumor cells, neutrophil phenotypic heterogeneity, the immune landscape of the TME and the nature of NET-inducing stimuli. Recognizing this paradox is essential for accurately understanding the role of NETs in CRC and for developing context-dependent therapeutic strategies.

Therefore, this review aims to systematically delineate the multifaceted roles and molecular mechanisms of NETs in CRC malignancy, with particular emphasis on the paradoxical interplay between their tumor-promoting and tumor-suppressive functions. We further summarize recent advances in CRC-specific molecular mechanisms of NETosis, NET biomarkers, detection and quantification methods, and therapeutic strategies targeting NETs, thereby providing an integrated framework and offering new perspectives for precision prevention and treatment of CRC.

## 2 Formation of NETs, core regulatory molecules, and biomarkers

### 2.1 Suicidal versus vital NETosis

NETs formation can occur through suicidal NETosis or vital NETosis. NETosis in CRC predominantly resembles suicidal NETosis, while evidence for vital NETosis remains limited and context-dependent.

Suicidal NETosis is initiated by potent agonists, including phorbol esters, microbial products, inflammatory mediators, and immune complexes. This process is triggered by the engagement of surface receptors on neutrophils, which subsequently activates downstream intracellular signaling cascades. Activated neutrophils generate large amounts of ROS via the NOX complex. ROS trigger calcium influx and subsequently activate PAD4, which catalyzes the conversion of arginine residues within histones to citrulline, yielding citrullinated histone H3 (CitH3). This reaction neutralizes the positive charges on histones, weakening their DNA-binding capacity and allowing chromatin to decondense and exit the nucleus into the cytoplasm. NE (neutrophil elastase) and MPO (myeloperoxidase) are subsequently discharged into the cytosol, where they interact with the unwound chromatin and potentiate its further decondensation. The final event involves disruption of both the nuclear and cytoplasmic membranes, which releases the restructured chromatin and bound proteins into the extracellular environment to generate NETs, with concurrent neutrophil lysis (14–16).

Unlike the lytic form, vital NETosis is a swift, lytic-independent process wherein neutrophils, upon stimulation with specific inflammatory mediators or complement proteins, rapidly deploy NETs while sustaining cellular integrity. This process may be NOX-independent, with calcium signaling directly activating PAD4, thereby inducing histone citrullination and chromatin decondensation. The released DNA derives primarily from mitochondria rather than the nucleus. The decondensed chromatin and granular proteins are packaged into intracellular vesicles, which are subsequently discharged from the cell to generate NETs. Importantly, this extrusion mechanism maintains the neutrophil’s membrane integrity, thereby preserving both its viability and chemotactic function post-NET release (14, 17–21). Furthermore, studies have revealed that the pore-forming activity of the pyroptosis-executing protein Gasdermin D is critical in certain forms of NETosis, thereby linking the pyroptosis and NETosis pathways (22). Together, these general modes of NET formation provide a mechanistic framework for understanding how CRC-specific signals induce and regulate NETosis in the TME.

### 2.2 CRC-specific molecular mechanisms of NETosis

Within this general mechanistic framework, NETosis in CRC is not merely a recapitulation of pathogen-induced neutrophil activation, but is driven by CRC-specific inflammatory, microbial, stromal and therapeutic stimuli within the TME. In CRC liver metastasis, a positive feedback loop operates between tumor-derived interleukin-8 (IL-8) and NET formation: NETs boost IL-8 expression in cancer cells, while IL-8 conversely triggers neutrophil activation and further NET release (23). SKAP1, when expressed in cancer cells, drives CRC progression through activation of the nuclear factor of activated T cells c1 (NFATc1)/C-X-C motif chemokine ligand 8 (CXCL8) axis, which in turn facilitates neutrophil recruitment and augments NET formation (24). Beyond tumor cells, inflammatory cancer-associated fibroblasts (CAFs) induced by fibroblast growth factor 19 represent a key stromal source of NET-promoting signals, driving both NET formation and CRC liver metastasis (25). In addition to dysbiosis, *Fusobacterium nucleatum* drives CRC-associated NETosis by engaging Toll-like receptor 4 (TLR4)-mediated ROS production and nucleotide-binding oligomerization domain-containing proteins 1/2 (NOD1/2) signaling pathways in neutrophils, which in turn fuels CRC progression and metastasis (26). Exosomal oncogenic Kirsten rat sarcoma viral oncogene (KRAS) has been shown to promote neutrophil extracellular trap formation in CRC by inducing IL-8 expression in tumor cells, thereby linking tumor genetic alterations with neutrophil activation and NETosis in the systemic circulation (27). Secreted protein acidic and rich in cysteine (SPARC) has been identified as a brake on NETosis in colorectal cancer metastasis; its deficiency relieves this suppression by activating the nicotinamide adenine dinucleotide phosphate oxidase (NOX) pathway, further corroborating NOX-dependent ROS generation as a core pathway in CRC-associated NETosis. Collectively, these CRC-specific signals converge on neutrophil activation signaling, ultimately resulting in NETs formation within the TME (28) (**Figure 1**).

**Figure 1:**
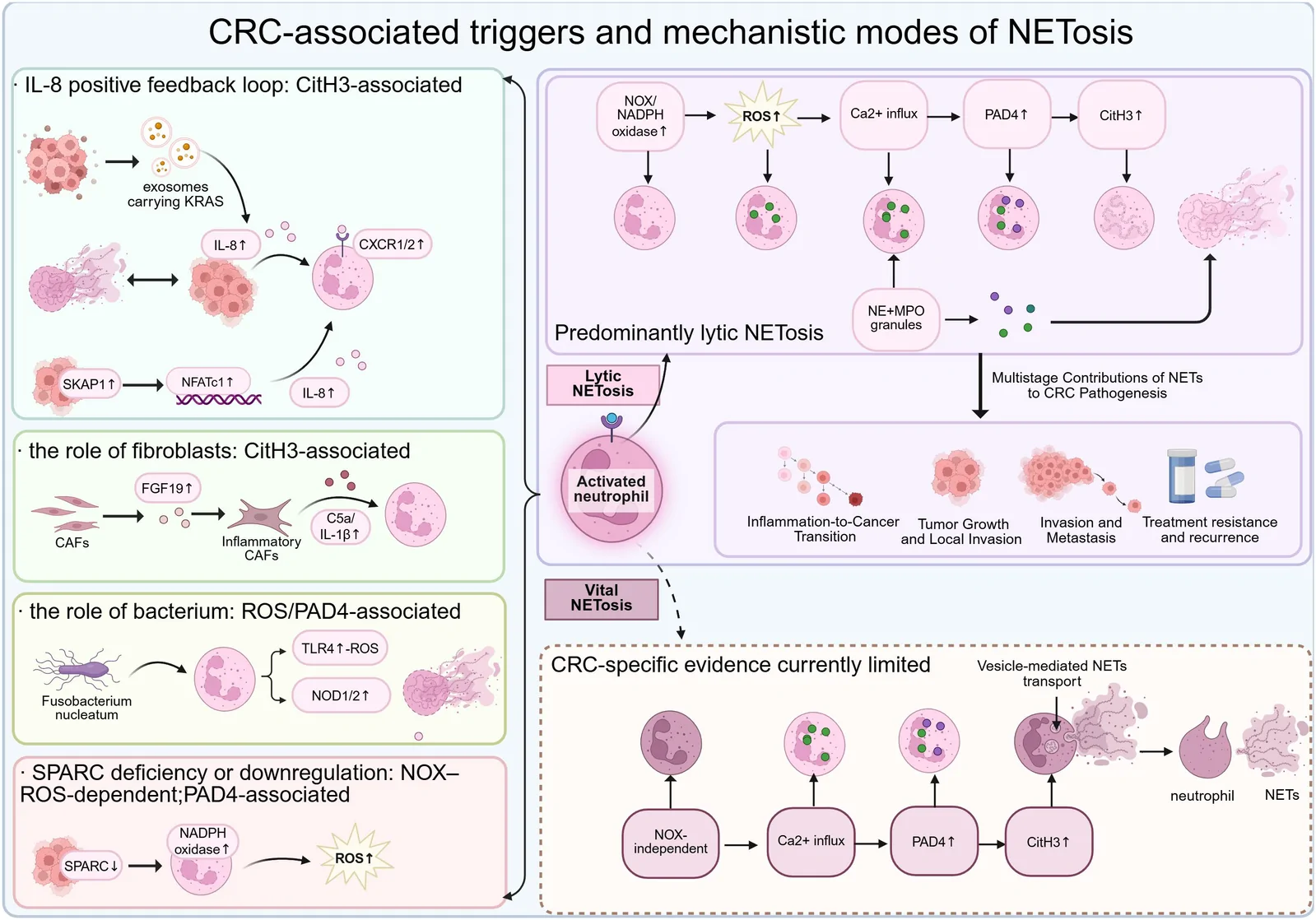
CRC-associated triggers and mechanistic modes of NETosis.

This figure summarizes CRC-specific pathways that induce NET formation and their predominant association with lytic NETosis. Specifically (1), tumor-derived inflammatory signaling: the IL-8 positive feedback loop and the SKAP1-NFATc1/CXCL8 axis promote neutrophil recruitment and CitH3-associated NET formation, while exosomal mutant KRAS further enhances IL-8 production; (2) stromal signaling: FGF19-induced inflammatory CAFs release C5a and IL-1β, promoting CitH3-associated NET formation; (3) microbial signaling: *Fusobacterium nucleatum* induces NET formation through ROS/PAD4-associated TLR4 signaling and NOD1/2 signaling; and (4) negative regulation: SPARC deficiency or downregulation promotes NET formation through NOX-ROS-dependent and PAD4-associated mechanisms. Collectively, these pathways are mechanistically consistent with predominantly lytic NETosis, involving ROS generation, PAD4 activation, histone citrullination, chromatin decondensation, and NET release. “Associated” indicates mechanistic consistency rather than directly demonstrated pathway dependence. The vital NETosis cascade shown within the dashed box is based primarily on mechanistic evidence from infection-related and other non-CRC studies. Direct experimental evidence supporting this pathway in CRC remains limited; therefore, it is presented as a conceptual alternative rather than an established CRC-specific mechanism. NETs subsequently contribute to multiple stages of CRC pathogenesis.

CAFs, cancer-associated fibroblasts; CitH3, citrullinated histone H3; CRC, colorectal cancer; C5a, complement component 5a; CXCR1/2, C-X-C chemokine receptor 1/2; FGF19, fibroblast growth factor 19; iCAFs, inflammatory cancer-associated fibroblasts; IL-1β, interleukin-1 beta; IL-8, interleukin-8; KRAS, Kirsten rat sarcoma viral oncogene; MPO, myeloperoxidase; NADPH, nicotinamide adenine dinucleotide phosphate; NE, neutrophil elastase; NETs, neutrophil extracellular traps; NETosis, neutrophil extracellular trap formation; NFATc1, nuclear factor of activated T cells 1; NOD1/2, nucleotide-binding oligomerization domain-containing proteins 1/2; NOX, NADPH oxidase; PAD4, peptidylarginine deiminase type 4; ROS, reactive oxygen species; SKAP1, Src kinase-associated phosphoprotein 1; SPARC, secreted protein acidic and rich in cysteine; TLR4, Toll-like receptor 4.

### 2.3 Core regulatory molecules and biomarkers

The formation, presence, and function of NETs are dictated by their core molecular components. These molecules not only serve as keys to understanding the mechanisms of NETosis but are also being translated into highly promising clinical biomarkers due to their detectability in the bodily fluids or tissues of CRC patients, facilitating prognostic assessment, therapeutic response prediction, and treatment monitoring (**Table 1**).

**Table 1:** Key regulatory molecules and biomarkers of NETs in CRC.

Biomarker	Biological relevance to NETs	Detection/performance in CRC	Clinical implication	Limitation	Ref.
PAD4	Core enzyme mediating histone citrullination and chromatin decondensation during NETosis	Upregulated in CRC tissues and peripheral blood	Reflects NET activation and serves as a potential therapeutic target	Clinical assays for PAD4 activity remain limited	(15, 29),
cfDNA	Major extracellular DNA component, partly originating from NET release	Elevated in serum/plasma of CRC patients	Candidate biomarker of circulating NET burden and adverse outcome	Limited specificity owing to interference from ctDNA and other DNA sources	(30, 32, 33)
MPO-DNA/NE-DNA complexes	Complexes formed by neutrophil granular proteins and NET-associated DNA	Mainly measured by ELISA; increased in CRC, particularly after surgery	Relatively specific circulating NET markers associated with recurrence and poor RFS	Assay methods and cutoff values have not been standardized	(35, 36),
CitH3	Direct product of PAD4 activity and a highly specific marker of NET formation	Detected by IHC in tumor tissue or ELISA in serum; elevated in CRC	Associated with systemic inflammation, advanced stage, chemoresistance, recurrence, metastasis, and poor prognosis	Further standardization across platforms and sample types is needed	(33, 36, 38)
NET-related gene signature/score	Integrative indicator of NET activity and its interaction with the tumor microenvironment	Derived from transcriptomic datasets such as TCGA/Xena; associated with clinicopathological characteristics	Useful for prognostic stratification and prediction of chemotherapy and immunotherapy benefit	Current evidence is mainly retrospective and requires prospective validation	(39)

cfDNA, cell-free DNA; CitH3, citrullinated histone H3; CRC, colorectal cancer; ELISA, enzyme-linked immunosorbent assay; IHC, immunohistochemistry; MPO, myeloperoxidase; NE, neutrophil elastase; NETs, neutrophil extracellular traps; PAD4, peptidylarginine deiminase type 4; RFS, recurrence-free survival; TCGA, the Cancer Genome Atlas.

#### 2.3.1 PAD4

During NET formation, PAD4 mediates the citrullination of histones, a modification that triggers chromatin decondensation (15). Targeting PAD4 through genetic or pharmacological approaches markedly suppresses NET formation in diverse preclinical models, positioning PAD4 as a viable therapeutic candidate (15). However, NET formation can also occur through PAD4-independent mechanisms, and clinical assays for measuring PAD4 activity have not yet been established (15). In a survey of several cancers including CRC, Chang et al. documented that PAD4 expression is upregulated in neoplastic tissues and circulating blood relative to normal controls (29).

#### 2.3.2 Cell-free DNA

cfDNA refers to fragmented DNA present in the circulation that originates from various sources, with DNA released from NETs constituting a major contributor (30, 31). In patients with CRC, quantification of cfDNA in serum samples has demonstrated that increased NETs production may correlate with carcinogenesis, postoperative complications, prolonged recovery times, and adverse clinical outcomes (32, 33). However, cfDNA is also abundantly derived from apoptotic tumor cells, namely circulating tumor DNA (ctDNA). Therefore, distinguishing the source of cfDNA remains a current research challenge, and the use of cfDNA alone lacks specificity for assessing NETs (34).

#### 2.3.3 MPO-DNA or NE-DNA complexes

NE and MPO, abundant in neutrophil granules, are recruited to the nucleus during NETosis to drive chromatin decondensation and become immobilized within the NET framework as core functional proteins (15). In CRC, measuring MPO or NE protein levels alone in the blood lacks sufficient specificity. Consequently, detecting stable complexes formed between MPO or NE and DNA (MPO-DNA/NE-DNA) is considered a more specific serological approach for quantifying the circulating NETs burden. Tohme S et al. studied 50 patients with metastatic colorectal cancer (mCRC) undergoing hepatectomy, collected postoperative serum samples, and measured MPO-DNA complex levels for comparison with healthy controls. Major hepatectomy was associated with a marked increase in NET formation relative to minor resection or healthy volunteer status, and this elevation independently correlated with higher risks of postoperative recurrence and mortality (*P* = 0.011) (35). Preoperative serum MPO-DNA complexes, as measured by enzyme-linked immunosorbent assay (ELISA) in the study by Okamoto et al., emerged as a significant correlate of reduced recurrence-free survival (36).

#### 2.3.4 CitH3

As a direct product of PAD4 activity, CitH3 is one of the most specific biomarkers for detecting NETs in tissues or blood (37). As a tissue-based marker, CitH3 detected by immunohistochemical serves as the pathological benchmark for NET identification, and its serum levels are not only elevated in CRC patients versus controls but also correlate with systemic inflammation and clinical stage (33). In a cohort of 146 CRC patients and 49 healthy controls, Wang et al. quantified serum CitH3 by ELISA and found that higher levels were associated with an unfavorable prognosis and diminished chemosensitivity (38). Furthermore, CitH3 expression in CRC tumor tissues has been associated with postoperative recurrence and metastasis (36).

#### 2.3.5 NETs-related gene signature models

Single biomarkers may not fully capture the complex roles of NETs within the TME. In recent years, constructing comprehensive signature models based on multi-omics data has emerged as a new trend. In 2025, Wu XL et al. obtained gene expression profiles of the Cancer Genome Atlas (TCGA)-CRC from the Xena database and developed a 10-gene NETs-related signature. This NETs signature correlated with the TME and clinicopathological characteristics, with lower scores correlating with more favorable prognosis. Further analyses demonstrated that this NETs-related signature not only predicted chemotherapeutic responses but also distinguished patients who would potentially benefit from immunotherapies like anti-programmed death-ligand 1(PD-L1) (39).

## 3 Multistage contributions of NETs to CRC pathogenesis

NETs play a contributory role across the full spectrum of CRC development and progression, encompassing the inflammation-to-cancer transition, CRC progression, promotion of metastasis, and CRC-related comorbidities (**Figure 2**).

**Figure 2:**
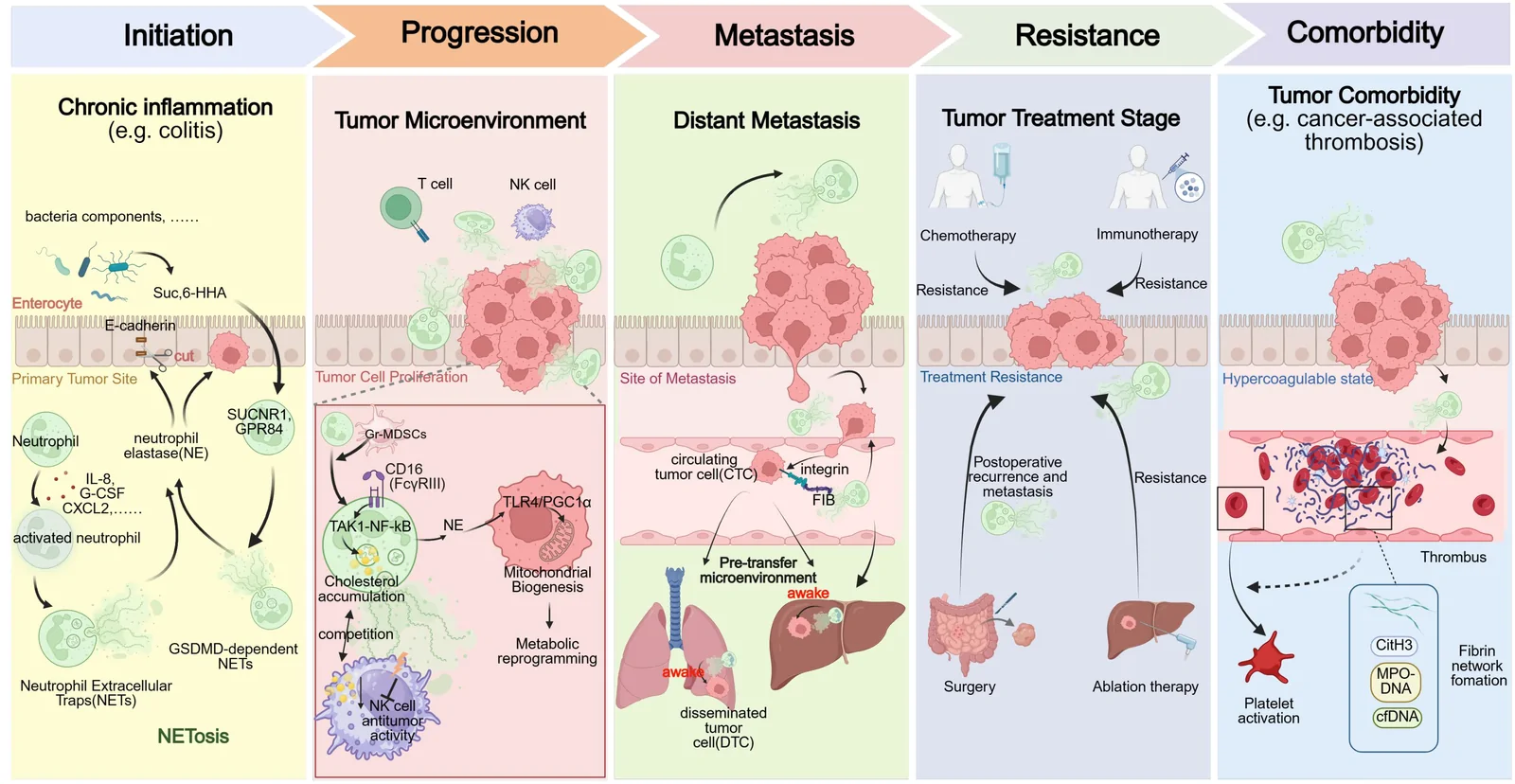
Multifaceted pro-tumorigenic roles of NETs across the full spectrum of CRC pathogenesis.

This figure illustrates the stage-specific contributions of NETs to CRC development, organized across five disease phases. (1) Initiation: In chronic colitis, NETosis is triggered via SUCNR1/GPR84 and GSDMD-dependent pathways. NETs disrupt the epithelial barrier through NE-mediated E-cadherin cleavage, promoting bacterial translocation and inflammation-to-cancer transition. (2) Progression: At the primary tumor site, CD16^+^ neutrophils activate TAK1-NF-κB signaling, driving cholesterol accumulation and metabolic reprogramming. NETs also activate TLR4/PGC1α-mediated mitochondrial biogenesis, enhancing tumor cell proliferation, while forming physical barriers that impede cytotoxic T cells and NK cells. (3) Metastasis: NETs capture CTCs via integrin interactions, remodel the pre-metastatic niche, and awaken dormant DTCs at distant organs. (4) Resistance: Chemotherapy, immunotherapy, surgery, and ablation therapy paradoxically induce NETosis, which promotes treatment resistance, immune evasion, and postoperative recurrence. (5) Comorbidity: NETs contribute to hypercoagulability and thrombosis by scaffolding platelet activation and fibrin deposition.

CitH3, citrullinated histone H3; cfDNA, cell-free DNA; CRC, colorectal cancer; CTC, circulating tumor cell; CXCL2, C-X-C motif chemokine ligand 2; DTC, disseminated tumor cell; FcγRIII, Fc gamma receptor III; FIB, fibrinogen/fibrin; G-CSF, granulocyte colony-stimulating factor; GPR84, G protein-coupled receptor 84; Gr-MDSCs, granulocytic-myeloid-derived suppressor cells; GSDMD, gasdermin D; IL-8, interleukin-8; MPO, myeloperoxidase; NE, neutrophil elastase; NETs, neutrophil extracellular traps; NF-κB, nuclear factor kappa-light-chain-enhancer of activated B cells; NK, natural killer; PGC1α, peroxisome proliferator-activated receptor gamma coactivator 1-alpha; Suc-6-HHA, succinyl-6-hydroxyhexanoic acid; SUCNR1, succinate receptor 1; TAK1, transforming growth factor-β-activated kinase 1; TLR4, Toll-like receptor 4.

### 3.1 Initiation phase: NETs drive inflammation-to-cancer transition

Chronic inflammation is a major driver of CRC, with approximately 20% of CRC cases arising from inflammatory bowel disease (IBD) (40). Within the inflammation-to-cancer transition from IBD to CRC, NETs act as both effectors and amplifiers driving malignant transformation. Their role begins with the disruption of the intestinal epithelial physical barrier: in active IBD mucosa, NE on NETs directly cleaves tight junction proteins such as E-cadherin, leading to barrier loss and bacterial translocation, thereby triggering and sustaining a chronic inflammatory state that is difficult to resolve (41)and laying the groundwork for carcinogenesis. In animal models, inhibition of NET formation reduces colitis severity and subsequent tumor development (42–44). Gut microbial dysbiosis may contribute to this process. For example, the metabolite succinate produced by Parasutterella excrementihominis activates succinate receptor 1/G protein-coupled receptor 84 signaling in neutrophils and increases Gasdermin D-dependent NETosis, thereby aggravating experimental colitis and colitis-associated tumorigenesis (45). Proteases associated with NETs may further damage the intestinal epithelium. In addition to NE, neutrophil-derived serine proteases activate protease-activated receptor 2 in epithelial cells, leading to forkhead box protein A2-mediated autophagic dysfunction and increased cellular stress (46). In the azoxymethane/dextran sulfate sodium model of colitis-associated cancer, increased expression of the NET-associated components matrix metalloproteinase-9 (MMP-9) and NE has also been linked to angiogenesis and abnormal epithelial proliferation (47). Chemokine signaling may sustain neutrophil recruitment and activation in this setting. C-X-C motif chemokine ligand 2 (CXCL2) attracts neutrophils and upregulates MMP-9 and NE expression, whereas activated tumor-associated neutrophils secrete inflammatory mediators that in turn boost CXCL2 production (47).

Collectively, these observations imply that NETs drive the colitis-to-CRC transition through multiple mechanisms, including disruption of the epithelial barrier, perpetuation of local inflammation, protease-induced tissue damage, and remodeling of the surrounding microenvironment. However, much of the supporting evidence comes from experimental models, and the relative contribution of these mechanisms in patients remains to be established.

### 3.2 Progression phase: NETs drive tumor growth and local invasion

#### 3.2.1 Construction of an immunosuppressive microenvironment

NETs serve as a central hub in shaping the immunosuppressive TME, dismantling anti-tumor immunity through a dual mechanism involving physical sequestration and functional suppression. The reticulated DNA structure of NETs can physically encase tumor cells, acting like a “cocoon” that blocks direct contact between cytotoxic T lymphocytes or natural killer (NK) cells and their target cells, thereby rendering immune attack ineffective. Intravital microscopy has directly captured the process by which NETs interfere with immune cell-CRC cell interactions (48). Within the TME, a phenotypically distinct neutrophil subset, such as CD16^+^ neutrophils, exhibits profound functional dysregulation. By activating the CD16/transforming growth factor-β-activated kinase 1/nuclear factor kappa-light-chain-enhancer of activated B cells axis, these cells abnormally accumulate cholesterol and highly express the scavenger receptors CD36 and LRP1.Such neutrophils not only compete with NK cells for cholesterol uptake, thereby blunting their anti-tumor function, but also release NETs that further damage NK cells (49). SKAP1 upregulates the expression of the CXCL8 by activating NFATc1 within tumor cells, thereby potently recruiting neutrophils to the tumor site and inducing NETs formation. Blockade of CXCL8 or NFATc1 significantly abrogates this pro-tumorigenic axis. More importantly, the recruited cells include not only neutrophils but also substantial populations of myeloid-derived suppressor cells (MDSCs) (24).Co-culture of neutrophils with granulocytic MDSCs (Gr-MDSCs) *in vitro* markedly induces NETosis, suggesting that these immunosuppressive cells form a positive feedback network in the TME that mutually reinforces and amplifies immunosuppression (50).

#### 3.2.2 Enhancement of tumor cell-intrinsic malignant potential

Beyond altering the external microenvironment, NETs can directly endow tumor cells with enhanced invasive and survival capacities. Exosomes derived from KRAS-mutant CRC can induce NETs formation by mediating IL-8 activation, and these NETs in turn significantly enhance CRC cell adhesion, viability, invasion, and migration (27).

#### 3.2.3 Metabolic reprogramming

NETs can directly intervene in the energy metabolism of tumor cells, thereby fueling their rapid proliferation. Emerging evidence indicates that NET-derived NE stimulates TLR4 on tumor cells, consequently upregulating peroxisome proliferator-activated receptor gamma coactivator 1-alpha (PGC1α) expression (51). As a master regulator of mitochondrial biogenesis, PGC1α drives increases in mitochondrial mass, mtDNA content, and ATP generation (51). Tumor cells treated with NETs exhibit enhanced oxygen consumption rates, mitochondrial membrane potential, and fusion/fission dynamics (as reflected by altered protein expression of DRP-1 and MFN-2), presenting a hypermetabolic phenotype. This NET-induced enhancement of mitochondrial biogenesis, dependent on the TLR4/PGC1α axis, provides a robust bioenergetic foundation for rapid tumor growth and proliferation (51). In animal models, mice deficient in NET-forming capacity (e.g., PAD4 knockout) display slow tumor growth and generally down-regulated mitochondrial function-related parameters within tumor tissues, further corroborating the critical role of NETs in driving tumor metabolic reprogramming (51).

### 3.3 Metastatic phase: NETs promote distant dissemination

Metastasis remains the primary cause of mortality in CRC patients, and NETs are critically involved in this process by promoting circulating tumor cells (CTCs) seeding, preconditioning the metastatic microenvironment at distant sites, and reactivating dormant micrometastases, thereby orchestrating CRC dissemination through multiple pathways.

#### 3.3.1 Capture of CTCs

In the circulation, NETs can capture CTCs through their DNA-based extracellular structures. Fibrinogen and other adhesion molecules associated with NETs interact with integrins (such as β1-integrin) expressed on CTCs, promoting their retention within the vasculature. This interaction may increase CTC survival under blood flow conditions and facilitate subsequent attachment and extravasation at distant sites. Intravital imaging studies have further demonstrated the accumulation of CTC clusters on NET structures *in vivo*, supporting a role for NETs in the early steps of metastatic dissemination (52).

#### 3.3.2 Remodeling of the pre-metastatic niche

NETs contribute to metastatic niche formation beyond their role in trapping circulating tumor cells. NET structures have been detected in metastatic target organs, including the liver and lung, where they can modify the local microenvironment and facilitate tumor cell colonization. In addition to serving as physical scaffolds, NET-derived DNA can function as a chemotactic cue for tumor cells. Extracellular NET-DNA is sensed by tumor cells via the transmembrane receptor coiled-coil domain containing 25 (CCDC25), which subsequently triggers the integrin-linked kinase-β-parvin signaling axis and drives tumor cell chemotaxis toward NET-rich regions (53). Furthermore, the NET-rich pre-metastatic microenvironment is profoundly immunosuppressive. First, surface programmed death-1 (PD-L1) on NETs binds T-cell PD-1, thereby driving T-cell exhaustion and compromising their metabolic capacity. Clinical studies have confirmed that soluble PD-L1 levels rise in parallel with the NET marker MPO-DNA in postoperative serum from CRC patients with liver metastases, and that NETs generated by postoperative neutrophils directly induce exhaustion of human CD4+ and CD8+ T cells (54). Moreover, tumor-associated macrophage-derived exosomes can activate a signal transducer and activator of transcription 3 (STAT3)/interleukin-1 beta (IL-1β) positive feedback loop in tumor cells by delivering molecules such as fibrinogen-like protein 2. Through IL-1β-mediated tumor-neutrophil crosstalk, NET formation is robustly induced alongside enhanced tumor cell stemness, culminating in the establishment of an immunosuppressive, stemness-enriched metastatic niche (55). High expression of phosphataseof regenerating liver 3 in liver metastases has likewise been shown to be closely associated with neutrophil and MDSCs infiltration as well as NETosis levels, serving as a strong indicator of poor prognosis (50). Multiple factors converge to promote NETs formation, thereby collectively exacerbating the TME. For instance, *Fusobacterium nucleatum*, a gut bacterium associated with CRC, triggers NET formation via TLR4-ROS and NOD1/2 pathways, consequently accelerating metastasis through the promotion of angiogenesis and epithelial–mesenchymal transition (EMT) (26). Similarly, the oral commensal *Streptococcus salivarius* has been linked to CRC metastasis by eliciting neutrophil NET formation via the IRGM1–IQGAP1–Wnt5a cascade (56). Moreover, surgery-related stresses such as hepatic ischemia-reperfusion injury promote NETs formation and Gr-MDSC infiltration via activation of the hepatocyte serum glucocorticoid regulated kinase 1-STAT3-serum amyloid A signaling axis, creating favorable conditions for postoperative liver metastatic recurrence (57).

#### 3.3.3 Awakening of dormant disseminated tumor cells

Some disseminated tumor cells (DTCs) in distant organs may remain quiescent after primary tumor resection, later giving rise to late metastatic recurrence. Groundbreaking studies have revealed that NETs induced by stimuli such as surgical trauma or chronic inflammation act as a critical alarm signal that disrupt DTCs dormancy. Laminin, a key component of the extracellular matrix, is sequentially and specifically cleaved by NE and MMP-9 associated with NETs. The resulting protease-modified laminin fragments bind to integrin α3β1 on the surface of DTCs, potently activating downstream proliferative signaling pathways and driving dormant cells back into the active cell cycle, ultimately leading to clinically detectable metastases (58).

#### 3.3.4 Promoting of metabolic and phenotypic conversion

NETs can also directly act on tumor cells to confer stronger metastatic capacity. *In vitro* studies demonstrate that exposure of CRC cells to NETs enhances their migratory and invasive capabilities by triggering EMT, as reflected by reduced E-cadherin expression and elevated levels of N-cadherin and vimentin (59). Mechanistic investigations further reveal that NET exposure activates the phosphatidylinositol 3-kinase/protein kinase B signaling cascade in tumor cells and enhances the expression of the glycolytic enzyme lactate dehydrogenase A. Elevated lactate dehydrogenase A promotes glycolysis (i.e., the Warburg effect), providing energy for rapid cell movement, and its knockdown suppresses NET-induced EMT and metastasis (59).

### 3.4 Resistance phase: NETs mediate therapeutic resistance and recurrence

Conventional therapies, while attacking tumors, often inadvertently induce the formation of NETs. These NETs then serve as a protective shield for tumor and mediate resistance to chemotherapy, immunotherapy, and even surgery through multiple mechanisms.

#### 3.4.1 Mediation of chemotherapy resistance

Upon chemotherapy, tumor cells secrete IL-1β, which in turn activates neutrophils to release NETs. These NETs, carrying integrin αvβ1, bind latent transforming growth factor-β (TGF-β) and employ MMP-9 to convert it into its bioactive form. Activated TGF-β promotes EMT in tumor cells-a process that is closely linked to chemoresistance (60). Chemotherapeutic drugs including oxaliplatin can elicit NETosis, resulting in markedly elevated NET levels in patients. However, the impact of NETs on treatment response is not uniformly detrimental and appears to be context-dependent. For instance, combining the glutaminase inhibitor CB-839 with 5-fluorouracil preferentially upregulates IL-8 expression in PIK3CA-mutant CRC, which recruits neutrophils, elevates their ROS production, and subsequently drives NET formation. In this specific therapeutic context, increased intratumoral NETs levels paradoxically correlate with longer progression-free survival. Mechanistic analysis revealed that cathepsin G (CTSG) carried by NETs can enter tumor cells through the receptor for advanced glycation endproducts(RAGE), subsequently inducing mitochondrial pathway-mediated apoptosis (61). This observation emphasizes the inherent heterogeneity of NET components and their biological functions, suggesting that the ultimate impact of NETs on therapeutic outcomes is likely governed by both the genetic makeup of the tumor and the specific drug regimen employed.

NETs also contribute to chemotherapy-related complications. In chemotherapy-induced peripheral neuropathy models, NET accumulation in dorsal root ganglia and peripheral nerves disrupts microcirculation and induces local ischemia, provoking mechanical hyperalgesia. Notably, NET degradation by deoxyribonuclease I (DNase I) or inhibition of MPO/PAD4 effectively relieves these neuropathy symptoms (62).

#### 3.4.2 Immunotherapy resistance

NETs represent a critical factor driving resistance to immune checkpoint inhibitors (ICIs). NETs form an immunosuppressive barrier within the TME through both physical and molecular strategies. The reticular NET architecture physically hinders cytotoxic T cel-tumor cell interactions, while PD-L1 displayed on NETs engages PD-1 on T cells and transmits inhibitory signals that drive T-cell exhaustion and metabolic impairment. Accordingly, NET degradation-using DNase I or the endogenous nuclease DNASE1L3-has been shown to restore CD8^+^ T-cell function and potentiate anti-PD-(L)1 therapy (63). A recent study has demonstrated that disrupting the fibroblast growth factor 19/extracellular signal-regulated kinase/IL-8 axis to suppress NET formation substantially enhances the efficacy of immune checkpoint blockade in microsatellite-stable (MSS) CRC (64). Inhibition of this pathway attenuates NET accumulation, mitigates the immunosuppressive TME, promotes CD8^+^ cytotoxic T-cell infiltration, and enhances the response of MSS CRC to anti-PD-1 therapy. These findings underscore the therapeutic promise of targeting stromal–neutrophil crosstalk to circumvent immunotherapy resistance.

#### 3.4.3 Perioperative metastatic risk

Although surgery is a curative intervention, the profound stress response it triggers can inadvertently create conditions favorable for tumor recurrence. Surgical injury triggers extensive release of damage-associated molecular patterns including interleukin-6 and high mobility group box 1, which potently stimulate NETosis and establish the perioperative period as a critical time window for NET generation. Prospective clinical studies have confirmed that postoperative plasma NET levels are substantially elevated in CRC patients, and this elevation independently correlates with reduced disease-free survival (35). Mechanistic insights from animal models have further revealed that surgical stress or NET exposure activates the MYC oncogenic pathway and upregulates fatty acid oxidation (FAO) programs in tumor cells. Specifically, NETs promote the uptake of long-chain fatty acids in tumor cells, partly via CD36 upregulation, and enhance FAO, thereby providing a vital energy source during circulation and metastatic colonization, especially under anoikis stress. Notably, pharmacological FAO inhibition effectively suppresses both surgery- and NET-driven metastatic growth. This mechanism is corroborated by patient data showing that the proliferative capacity of postoperative plasma correlates with NET levels and CD36 expression (65).

#### 3.4.4 Impact of cryoablation

Beyond chemotherapy and surgery, local interventions such as cryoablation can also elicit a sustained neutrophilic inflammatory response, promote the N1-to-N2 phenotypic switch, and induce PAD4-dependent NETosis through intracellular calcium elevation, collectively fostering post-treatment immune evasion. Interruption of neutrophil chemotaxis via the CXCL2-C-X-C Motif Chemokine Receptor 2 axis or direct NET degradation can restore anti-tumor immune activity (66).

### 3.5 Comorbidity phase: NETs and CRC-associated comorbiditie

Patients with CRC frequently present with a range of comorbidities, among which cancer-associated thrombosis is a leading cause of non-cancer mortality. NETs serve as a central bridge connecting tumor biology to a hypercoagulable state. Clinical studies have demonstrated that the incidence of venous thromboembolism (VTE) in CRC patients can be as high as 17.53%, and that patients who develop VTE exhibit significantly elevated plasma levels of NET-specific markers such as CitH3, MPO-DNA, and cfDNA, which act as independent biomarkers for predicting thrombotic risk (67). The procagulant activity of NETs is mediated through multiple synergistic mechanisms. The DNA scaffold acts as a negatively charged surface to support the coagulation cascade and directly activate factor XII. NETs released by TANs express tissue factor, thereby potently triggering the extrinsic coagulation pathway. Moreover, NET-resident histones (e.g., CitH3) activate platelet Toll-like receptors 2 and 4, resulting in platelet activation and aggregation. Activated platelets in turn further stimulate NETs release, establishing a prothrombotic NET-platelet positive-feedback loop (68). Furthermore, studies have shown that NETs levels correlate with markers of fibrinolytic system inhibition, suggesting a potential interference with fibrinolytic balance (67). Mechanistically, bioinformatic screening has identified Fc gamma receptor 1A (FCGR1A) as a key gene that is highly expressed in CRC patients and positively correlated with NETs level. Mechanistically, bioinformatic screening has identified FCGR1A as a key gene that is highly expressed in CRC patients and positively correlated with NETs levels. Importantly, inhibition of FCGR1A effectively reduces NETs formation, offering a new target for the development of anti-thrombotic therapies targeting NETs (67). Accordingly, profiling NET levels could help flag individuals at heightened VTE risk, and NET-targeted intervention may offer a fresh tactic for the prevention and management of cancer-associated thrombosis.

### 3.6 The paradoxical nexus of NETs in CRC: reconciling pro- and anti-tumor effects

A central premise of this review is that NETs in CRC do not act as an unequivocal pro-tumorigenic force; instead, their biological effect is dictated by a defined set of contextual variables. A growing body of evidence shows that the very same NET constituents can either enhance or constrain tumor growth, depending on (i) the tumor genetic background, (ii) the dominant neutrophil subset, (iii) the immune contexture, and (iv) the precipitating trigger (**Figure 3**). For example, in PIK3CA-mutant CRC, the pairing of the glutaminase inhibitor CB-839 with 5-fluorouracil preferentially elicits IL-8 and recruits neutrophils that release NETs loaded with CTSG; CTSG enters tumor cells via RAGE and triggers mitochondrial apoptosis, thereby prolonging progression-free survival in preclinical models (61). Conversely, in the same tumor type, surgery-induced NETs reprogram cancer-cell lipid metabolism through MYC/FAO signaling and accelerate metastatic outgrowth (65). These diametrically opposite outcomes underscore that the function of NETs is not intrinsic but is dynamically shaped by their milieu.

**Figure 3:**
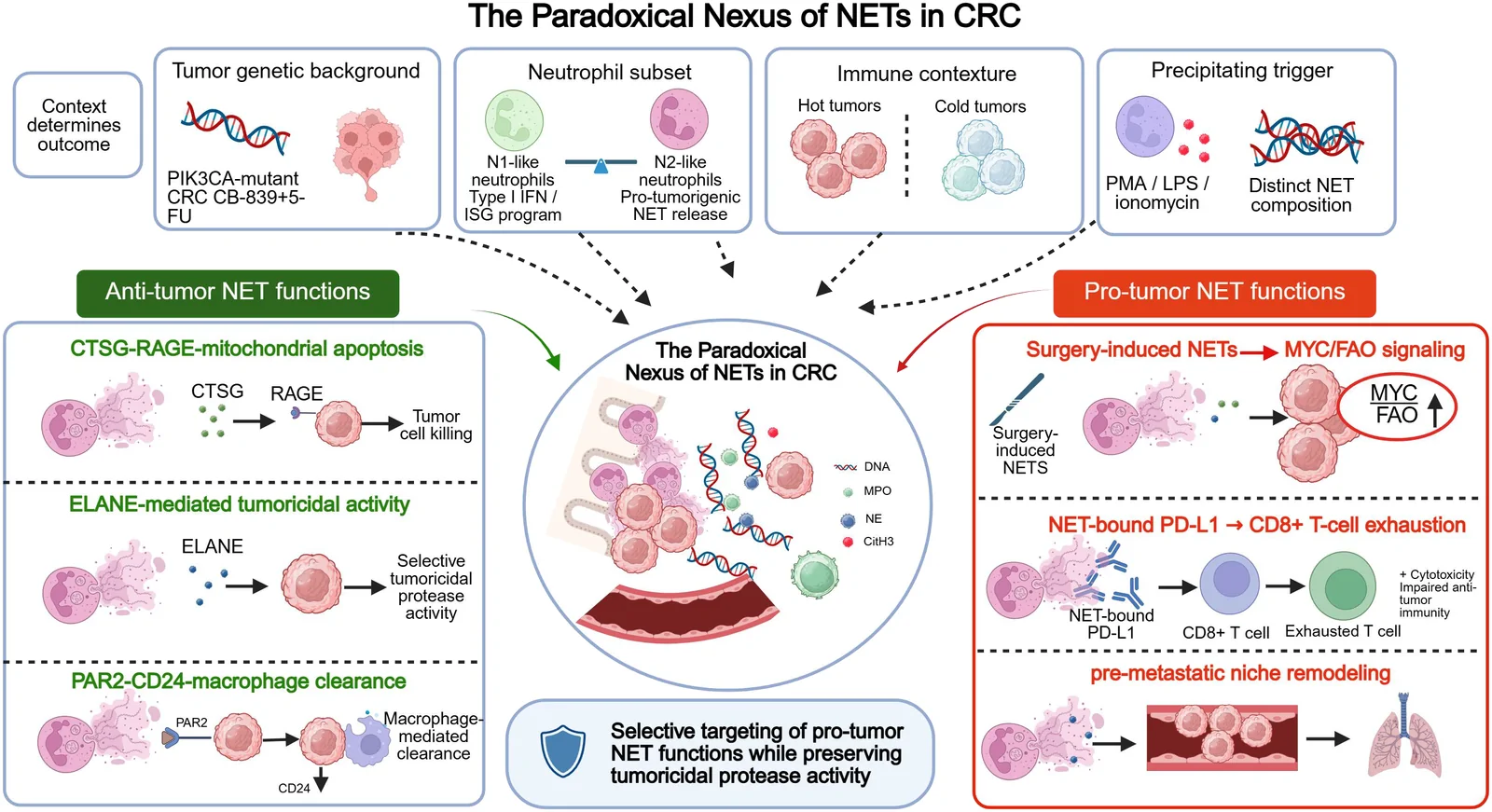
Schematic representation of the “paradoxical nexus” model describing the opposing effects of NETs in CRC and the factors influencing their biological consequences.

The figure summarizes the anti-tumor and pro-tumor functions of NETs and the major conditions that may determine their effects. (A) Anti-tumor NET functions: NET-associated components, including proteases such as ELANE and CTSG, may contribute to tumor cell killing or immune-mediated clearance under specific settings. (B) Pro-tumor NET functions: NETs can promote CRC progression through mechanisms involved in metastasis, immune suppression, and tumor-supportive signaling (see **Figure 2**). (C) Contextual determinants: The effects of NETs may vary according to tumor genetic background, neutrophil populations, immune environment, and the stimuli inducing NET formation. These factors influence whether NETs exert tumor-promoting or tumor-restrictive activities. Therefore, therapeutic strategies should focus on selectively interfering with disease-associated NET functions while maintaining physiological neutrophil-mediated defense mechanisms.

5-FU, 5-fluorouracil; CB-839, glutaminase inhibitor (telaglenastat); CD8^+^, cluster of differentiation 8; CD24, cluster of differentiation 24; CitH3, citrullinated histone H3; CRC, colorectal cancer; CTSG, cathepsin G; ELANE/NE, neutrophil elastase; FAO, fatty acid oxidation; IFN, interferon; ISG, interferon-stimulated gene; LPS, lipopolysaccharide; MPO, myeloperoxidase; MYC, MYC proto-oncogene; NETs, neutrophil extracellular traps; N1-like, anti-tumor neutrophil phenotype; N2-like, pro-tumor neutrophil phenotype; PAR2, protease-activated receptor 2; PD-L1, programmed death-ligand 1; PIK3CA, phosphatidylinositol-4,5-bisphosphate 3-kinase catalytic subunit alpha; PMA, phorbol 12-myristate 13-acetate; RAGE, receptor for advanced glycation endproducts.

Several NET-linked proteases have been tied to the tumor-suppressive effects of NETs under specific conditions. Cui et al. (69)showed that human NE can selectively induce cancer cell death while exhibiting limited toxicity toward non-transformed cells, suggesting that this classical NET component may retain intrinsic tumoricidal activity. In CRC, NE released from NETs has also been linked to direct tumor cell killing. In line with this, Li et al. showed that the CTSG/RAGE/mitochondrial-apoptosis axis underlies the anti-tumor activity of NETs when CB-839 is combined with 5-fluorouracil (61). These findings indicate that the biological consequence of NET formation depends not only on the amount of NETs produced but also on the molecular composition of NETs and the surrounding therapeutic context. In addition to direct tumor cell injury, NETs may also limit metastatic dissemination through innate immune mechanisms. Liu et al. recently reported that NET-associated serine proteases activate protease-activated receptor 2 on disseminated tumor cells, resulting in reduced expression of the phagocytic checkpoint CD24 and increased susceptibility to macrophage-mediated clearance. This ‘‘eat-me’’ switch renders metastasizing tumor cells susceptible to macrophage-mediated clearance, thereby impeding metastatic seeding in the early phase (70).

Three additional contextual layers reinforce the paradoxical nature of NETs in CRC. First, neutrophil-intrinsic plasticity is critical: N1-polarized neutrophils are reported to mount anti-tumor activity through type I interferon-stimulated gene programs, whereas N2-polarized, tumor-associated neutrophils preferentially release pro-tumorigenic NETs (71). Second, the immunological contexture of the TME determines the readout of NETs signaling: in T-cell-inflamed (“hot”) tumors, NET-bound PD-L1 dominates the response by inducing CD8+ T-cell exhaustion (54), whereas in immune-desert (“cold”) tumors, the dominant effect of NETs is structural trapping of tumor cells and remodeling of the pre-metastatic niche. Third, the kinetics and trigger of NETosis shape the resulting NETs composition: protein-content and histone-citrullination analyses demonstrate that phorbol 12-myristate 13-acetate-, lipopolysaccharide-, and ionomycin-induced NETs differ markedly in their proteomes, and that NETs released during chemotherapy or surgery carry distinct damage-associated molecular patterns (72). Together, these observations argue that NETs should be regarded as a context-dependent, dynamic nexus rather than a uniformly pro-tumor entity.

Clinically, this framework implies that NETs biomarkers must be interpreted in context (e.g., high levels may be prognostic or therapeutic depending on genotype and treatment), and that therapy should shift from broad NETs suppression to selective targeting of pro-tumorigenic NETs functions while preserving the tumoricidal protease arm.

## 4 Quantitative detection of NETs in CRC

Reliable NET detection in CRC is necessary to translate the paradoxical-nexus concept into clinical decisions. Available platforms include circulating biomarker assays, tissue imaging, flow cytometry (FCM), and multi-omics profiling (35–39, 73–79), complemented by emerging label-free virtual staining and artificial-intelligence-assisted image analysis (80, 81). Because no single method captures NET abundance, morphology, composition, and spatial context, orthogonal validation remains essential.

### 4.1 Circulating biomarker assays

Circulating NET burden is commonly estimated using ELISA for MPO-DNA, NE-DNA, or CitH3, fluorescent cfDNA assays, and polymerase chain reaction-based measurements (30, 35–38, 73). These scalable assays are useful for clinical cohorts but cannot reliably distinguish NET-derived material from DNA or proteins released by other processes. They should therefore be paired with morphological or tissue-based confirmation whenever possible.

### 4.2 Flow cytometry

FCM enables rapid, single-cell evaluation of NETosis with relatively objective and reproducible readouts. Masuda et al. quantified extracellular DNA using the membrane-impermeable dye SYTOX Green and reported agreement with microscopy-based measurements (R² = 0.73). Combining this dye with MPO immunostaining increased analytical specificity, as MPO was detected in approximately 76.1% of SYTOX Green-positive events (74). Multiparameter panels incorporating MPO and CitH3 have also been developed to identify NET-forming cells (75). Imaging FCM, represented by the ImageStream platform, further integrates fluorescence signals with changes in nuclear size and chromatin structure, facilitating high-throughput assessment and differentiation of suicidal from vital NETosis (76). Nevertheless, preparation of cell suspensions may exclude adherent NETotic cells or disrupt extracellular DNA aggregates, resulting in underestimation of NET formation. FCM results should therefore be interpreted together with complementary evidence from immunofluorescence, ELISA, or related assays.

### 4.3 Tissue-based histopathological detection

Immunohistochemistry (IHC) and multiplex IF of CitH3 and MPO on formalin-fixed paraffin-embedded CRC sections provide spatial information on intra-tumoral NETs density. A large multicenter cohort study (N = 1927) using multiplex IHC identified NETs showing positivity for CitH3 and granulocytes expressing CD66b, and quantified their densities in tumor tissues using image analysis and machine learning (77). Okamoto et al. performed IHC staining for MPO, CitH3, MMP9 and NE in primary CRC specimens and demonstrated that high intratumoral NETs expression correlates with unfavorable relapse-free and overall survival in CRC patients (36). Immunofluorescence staining of CitH3 and extracellular DNA has also been widely used to visualise NETs in CRC tissues (28).

### 4.4 Multi-omics signatures and integrative profiling

At the multi-omics level, several NET-related gene-signature models have been developed from TCGA-CRC data. A 10-gene NETs score constructed via LASSO regression predicts chemotherapy response and identifies CRC patients likely to benefit from ICIs (39). Through integrative single-cell and spatial transcriptomic profiling, Tang et al. identified two colon cancer subtypes driven by NETs and constructed a 4-gene prognostic signature (ARRDC1, TUBB2A, DUSP5, SLC2A3). The NETs-high subtype was characterized by enhanced oxidative phosphorylation and mitochondrial respiration, which in turn fostered immune evasion and therapy resistance (78). Jin et al. employed multi-omics analysis to screen three major NETs genes(TIMP1, F3, and CRISPLD2) and constructed a NETs score that not only predicts CRC patient prognosis but also reveals distinctions in microsatellite instability, checkpoint expression, and predicted responsiveness to PD-L1-directed therapy (79). Importantly, integrating circulating biomarkers with tissue-based and multi-omics readouts is expected to yield the most clinically informative NETs profile for individual CRC patients.

### 4.5 Artificial intelligence and emerging technologies

Beyond conventional tissue-based approaches, artificial intelligence (AI) is enabling NETs assessment to incorporate cellular morphology, spatial organization, and multimodal molecular information. The machine-learning-assisted analysis of CitH3-positive NETs in large CRC tissue cohorts described above illustrates the feasibility of computational quantification in clinical specimens (77).

At the cellular level, deep-learning models can identify NETosis-associated morphological changes that may not be adequately captured by conventional fluorescence-intensity thresholds. Elsherif et al. developed convolutional neural networks based largely on nuclear morphology, achieving greater than 94% accuracy in distinguishing NETotic from non-NETotic neutrophils. The models also separated NETosis from necrosis and recognized morphological patterns associated with different NET-inducing pathways (103). Such morphology-aware classifiers could complement marker-based measurements and reduce observer-dependent variation. Petchakup et al. subsequently introduced a label-free virtual-staining strategy in which a deep convolutional neural network transformed phase-contrast images of neutrophils captured in a microfluidic device into predicted fluorescence images resembling SYTOX Green staining. The resulting measurements showed a linear relationship with conventional fluorescence readouts and differentiated NETs phenotypes induced by lipopolysaccharide, phorbol ester, and calcium ionophore (80). Because this method avoids antibody labelling and preserves cells for downstream analysis, it may eventually support rapid functional assessment of circulating neutrophils from patients with CRC. In parallel, automated platforms such as NETQUANT permit high-throughput, single-cell quantification by integrating nuclear deformation, cell expansion, and DNA-associated fluorescence signals (81). However, NETQUANT should be regarded as an automated rule-based image-analysis system rather than a deep-learning model.

Whole-slide computational pathology represents another potentially scalable direction. Deep-learning cell classifiers have already been applied to routine hematoxylin–eosin-stained CRC sections to identify multiple immune and stromal populations and quantify their spatial relationships (104). Although these models do not yet detect NETs directly, they provide a technical foundation for future NETs-specific algorithms. Such systems could co-register hematoxylin–eosin images with multiplex immunofluorescence for CitH3, MPO or neutrophil elastase, extracellular DNA, and neutrophil markers, and then map NETs in relation to tumor cells, vessels, necrotic regions, and immune-cell niches. Imaging-derived features could also be integrated with circulating NETs markers, single-cell RNA sequencing, spatial transcriptomics, and computational NETs-activity scores (78). This multimodal framework may provide a more biologically informative assessment than any isolated marker.

Nevertheless, most AI-enabled approaches remain investigational. Their translation is constrained by the absence of a universally accepted NETs ground truth, limited specificity of individual markers, variability in tissue processing and imaging systems, and performance shifts between institutions. Future studies should therefore employ multi-marker annotations, harmonized imaging procedures, explainable model outputs, and prospective external validation in independent CRC cohorts. Addressing these challenges will be essential for developing AI-supported NETs measurements into reproducible biomarkers and therapeutic-response indicators in CRC.

## 5 Therapeutic strategies targeting NETs in CRC

Given the pivotal involvement of NETs in CRC progression, NET targeting has surfaced as a highly promising therapeutic avenue. Current tactics mainly encompass inhibiting NET formation, promoting NET clearance, blocking NET function, and combination regimens (**Figure 4** and **Table 2**).

**Figure 4:**
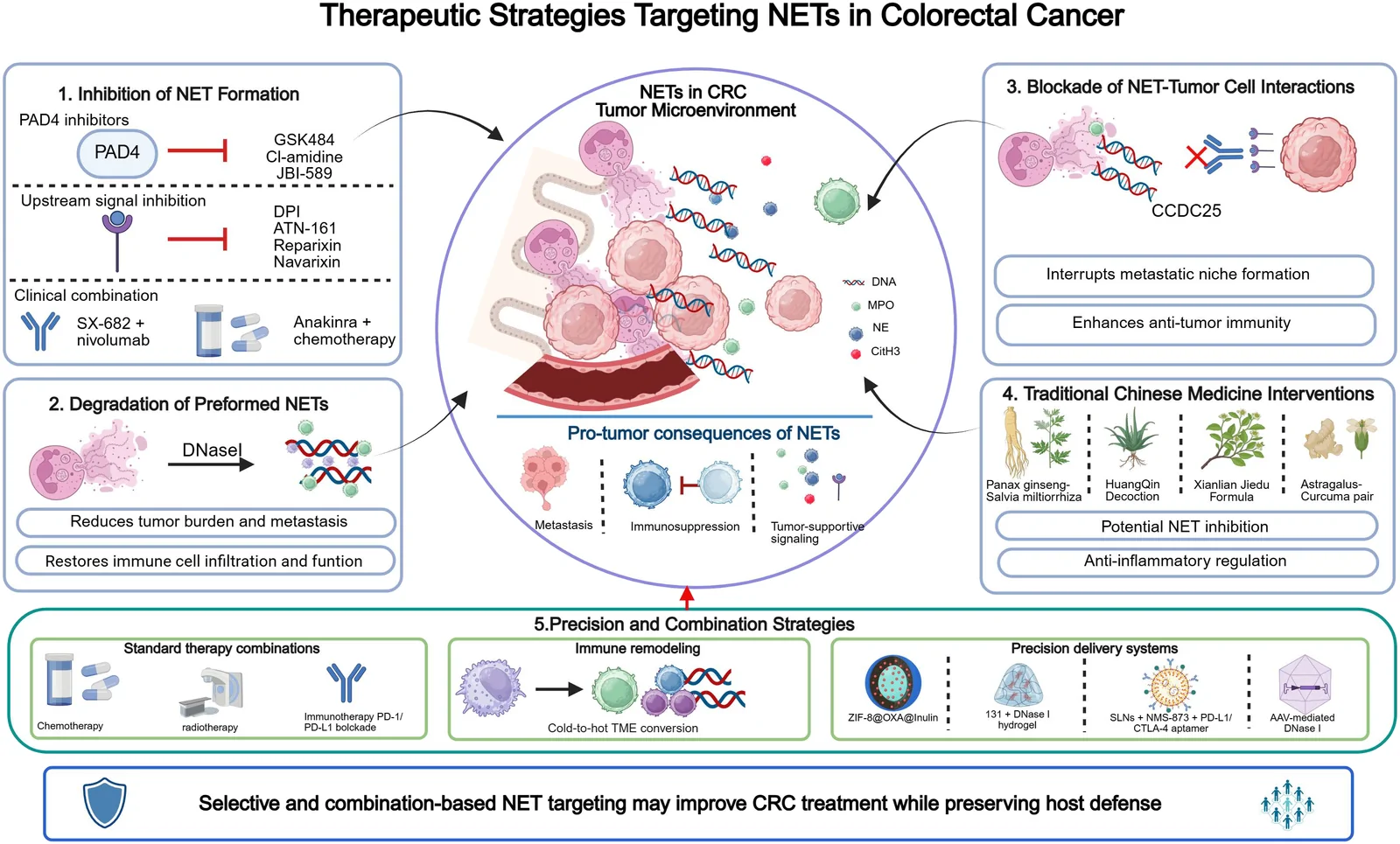
Therapeutic strategies targeting NETs in CRC.

**Table 2:** NET-targeted therapeutic strategies in CRC.

Strategy	Representative agents/approaches	Main mechanism	Evidence in CRC	Clinical implication/status	Ref.
Inhibition of NET formation: PAD4 inhibitors	GSK484; Cl-amidine; JBI-589	Suppress PAD4-dependent histone citrullination and NETosis	GSK484 enhanced radiosensitivity and inhibited NET formation *in vitro* and *in vivo*; Cl-amidine showed efficacy in UC and CRC prevention; JBI-589 inhibited PAD4-mediated neutrophil migration and tumor progression	PAD4 is a core therapeutic target for NET-directed intervention	(82–84)
Inhibition of upstream activating signals: ROS/NOX blockade	DPI combined with ATN-161	Inhibits ROS generation and integrin-mediated NET-associated prometastatic signaling	Combination treatment suppressed NET-mediated metastasis in CRC models	Promising preclinical strategy for limiting NET-driven dissemination	(28)
Inhibition of upstream activating signals: chemokine receptor antagonists	Reparixin; Navarixin; SX-682	Blocks CXCR1/2 signaling, thereby reducing neutrophil recruitment and NET induction	Reparixin reduced tumor growth and metastasis in preclinical CRC models; navarixin inhibited tumor growth, angiogenesis, and liver metastasis in murine CRC; SX-682 plus nivolumab is under clinical evaluation in RAS-mutant MSS mCRC	Represents a translational strategy linking neutrophil trafficking blockade with immunotherapy	(86–88)
Inhibition of inflammatory receptor signaling	Anakinra	Blocks IL-1 receptor-mediated inflammatory activation associated with neutrophil responses	Evaluated in combination with chemotherapy in advanced CRC	May complement conventional therapy by attenuating NET-promoting inflammation	NCT02090101
Degradation of preformed NETs	DNase I	Hydrolyzes the DNA backbone of NETs and dismantles existing NET structures	Sustained release of DNase I plus propranolol disrupted NETs, reduced microvascular density, modulated immune responses, and inhibited metastasis	Direct NET clearance is a rational strategy to counteract metastatic niche formation	(89)
Blockade of NET–tumor cell interaction	CCDC25-targeted inhibition	Interrupts tumor cell sensing of NET-derived DNA signals	Mechanistically proposed as a CRC-relevant strategy to block NET-driven metastatic signaling	A promising precision approach, but still at an early stage	(53)
Traditional Chinese medicine-based interventions	Ginseng–Salvia herb pair; Huangqin decoction; Xianlian Jiedu formula; Astragalus–Curcuma components	Modulate neutrophil function, oxidative stress, inflammation, and PAD4-dependent NETosis	Reported to inhibit NET formation, neutrophil infiltration, colitis-associated carcinogenesis, or CRC liver metastasis in experimental studies	Suggests potential value of multi-target integrative intervention against NET-driven CRC progression	(42, 90–92)
Combination strategies	GSK484 plus radiotherapy; DNase I plus anti-PD-1; ZIF-8@OXA@Inulin; omPEG-SLN/NMS-873/P1C4; AAV-DNase I	Enhances cytotoxicity, reverses immune exclusion, remodels gut microbiota and TME, or overcomes immunotherapy resistance	Preclinical studies showed improved radiosensitivity, synergistic anti-tumor immunity, and enhanced therapeutic efficacy in CRC	Combination therapy may maximize the translational value of NET-targeting in CRC	(51, 64, 82, 93, 95, 96)

AAV, adeno-associated virus; Anakinra, interleukin-1 receptor antagonist; ATN-161, integrin α5β1 inhibitor; CCDC25, coiled-coil domain containing 25; Cl-amidine, peptidylarginine deiminase 4 inhibitor; CRC, colorectal cancer; DNase I, deoxyribonuclease I; DPI, diphenyleneiodonium chloride; GSK484, peptidylarginine deiminase 4 inhibitor; ICIs, immune checkpoint inhibitors; JBI-589, peptidylarginine deiminase 4 inhibitor; mCRC, metastatic colorectal cancer; MSS, microsatellite-stable; Navarixin, CXCR2 antagonist; NMS-873, valosin-containing protein inhibitor; omPEG, orthogonal masked polyethylene glycol; OXA, oxaliplatin; P1C4, bispecific PD-L1/CTLA-4 aptamer; PD-1, programmed death-1; PD-L1, programmed death-ligand 1; Reparixin, CXCR1/2 inhibitor; ROS, reactive oxygen species; SLNs, solid lipid nanoparticles; SX-682, CXCR1/2 inhibitor; TCM, traditional Chinese medicine; UC, ulcerative colitis; ZIF-8, zeolitic imidazolate framework-8.

This figure summarizes current therapeutic approaches targeting NETs in CRC. Strategies include (1): inhibition of NET formation through PAD4 inhibitors or upstream signal blockers; (2) degradation of preformed NETs using DNase I; (3) blockade of NET-tumor cell interactions; (4) Traditional Chinese Medicine interventions with multi-target mechanisms; and (5) precision delivery platforms that enable localized NETs degradation and combination with chemotherapy, radiotherapy, or immunotherapy to overcome treatment resistance.

AAV, adeno-associated virus; ATN-161, integrin inhibitor; CCDC25, coiled-coil domain containing 25; CitH3, citrullinated histone H3; Cl-amidine, PAD4 inhibitor; CRC, colorectal cancer; CTLA-4, cytotoxic T-lymphocyte-associated protein 4; DNA, deoxyribonucleic acid; DNase I, deoxyribonuclease I; GSK484, PAD4 inhibitor; ^131^I, iodine-131; JBI-589, PAD4 inhibitor; MPO, myeloperoxidase; NE, neutrophil elastase; NETs, neutrophil extracellular traps; NK, natural killer; NMS-873, valosin-containing protein inhibitor; OXA, oxaliplatin; PAD4, peptidylarginine deiminase type 4; PD-1, programmed cell death protein 1; PD-L1, programmed death-ligand 1; PPI, protein-protein interaction;

ROS, reactive oxygen species; SLNs, solid lipid nanoparticles; SX-682, CXCR1/2 inhibitor; TME, tumor microenvironment; ZIF-8, zeolitic imidazolate framework-8.

### 5.1 Inhibition of NETs formation

#### 5.1.1 PAD4 inhibitors

PAD4 is currently the most intensively pursued target. The small-molecule inhibitor GSK484 has been shown to enhance the radiosensitivity of CRC and curb NETs formation both *in vivo* and *in vitro (*82). Cl-amidine has demonstrated efficacy in treating ulcerative colitis and preventing CRC (83). Furthermore, a new selective inhibitor, JBI-589, blocks PAD4-mediated neutrophil migration to restrain tumor progression (84). Beyond direct PAD4 inhibition, drug repurposing has recently emerged as an alternative strategy for suppressing NETosis. Indacaterol, an approved long-acting β2-adrenergic receptor agonist. In preclinical CRC models, indacaterol markedly reduced NET accumulation, suppressed liver metastasis, and prolonged survival (85).

#### 5.1.2 Inhibition of upstream activation signals

Aside from directly blocking the core enzymatic machinery of NETosis, intercepting the signaling cascades that trigger neutrophil recruitment and activation represents a parallel therapeutic avenue. These upstream signals-ranging from ROS and chemokine gradients to inflammatory cytokines-collectively orchestrate the pathological accumulation of neutrophils within the TME and their subsequent NET release. One major class of upstream inhibitors targets the NOX/ROS axis, which is a central driver of suicidal NETosis. The NOX inhibitor diphenyleneiodonium chloride, when combined with the integrin α5β1 antagonist Ac-PHSCN-NH2, has demonstrated efficacy in curbing NET-mediated metastatic spread in CRC models, suggesting that dual blockade of ROS generation and integrin-dependent adhesion may offer synergistic benefit (28). However, the clinical translatability of NOX inhibitors remains constrained by concerns over broad immunosuppressive effects, as ROS signaling is also critical for normal antimicrobial host defense. Another extensively investigated upstream node is the IL-8/CXCR1/2 chemokine axis, which serves as a principal chemoattractant for neutrophil trafficking into tumor sites. Several small-molecule antagonists have been developed to disrupt this signaling. For instance, the oral CXCR1/2 inhibitor reparixin has been shown to impede IL-8-mediated neutrophil infiltration, thereby attenuating tumor growth and metastatic burden in preclinical settings (86). Similarly, the CXCR2-selective antagonist navarixin (SCH-527123) has exhibited anti-tumor activity and suppression of liver metastasis in murine CRC models, and has also been reported to sensitize tumor cells to oxaliplatin (87). Extending these observations into the clinical arena, A Phase Ib/II trial (NCT02823401) is ongoing to evaluate navarixin combined with PD-1 blockade in patients with various advanced solid tumors, including CRC. The ongoing Phase I/II STOPTRAFFIC-1 trial (NCT04599140) is investigating the CXCR1/2 inhibitor SX-682 combined with nivolumab in patients with RAS-mutant, MSS metastatic CRC (88). These trials reflect growing interest in combining neutrophil-directed strategies with immune checkpoint inhibitors, particularly in immunologically “cold” tumors where neutrophil-driven immunosuppression is a major barrier to efficacy. Beyond chemokine receptor blockade, interventions targeting inflammatory mediators upstream of neutrophil activation have also been pursued. The IL-1 receptor antagonist anakinra, which attenuates IL-1β-driven inflammatory signaling, has been investigated in combination with chemotherapy for advanced CRC (NCT02090101). While the rationale for such combinations is mechanistically sound-given that IL-1β is a potent inducer of NETosis-clinical data remain preliminary, and the optimal patient selection criteria have yet to be defined.

### 5.2 Degradation of preformed NETs

DNase I directly hydrolyzes the DNA backbone of NETs. Studies show that sustained release of DNase I combined with propranolol disrupts NETs and antagonizes catecholamines, thereby reducing micro-vascular density, blocking MDSCs, suppressing the secretion of multiple pro-inflammatory cytokines, enhancing NK-cell function, and inhibiting cytotoxic T-cell exhaustion, ultimately suppressing tumor metastasis (89).

### 5.3 Blockade of NET-tumor cell interactions

Another promising strategy is to develop agents that block receptors on tumor cells responsible for recognizing NETs. For instance, inhibitors targeting the CCDC25 receptor could theoretically abrogate the capacity of tumor cells to perceive NET-DNA signals, offering a precise and highly promising approach (53).

### 5.4 Potential value and exploration of traditional chinese medicine interventions

Traditional Chinese Medicine (TCM) possesses holistic and multi-target advantages in regulating the imbalance between inflammation and immunity. Certain herbal formulas or active monomers with heat-clearing, blood-cooling, blood-activating and detoxification may influence NETosis by modulating neutrophil function, anti-oxidative stress, and anti-inflammatory pathways. The herbal pair Panax ginseng-Salvia miltiorrhiza and its bioactive components exert anti-metastatic effects against malignancies by inhibiting CD62E-dependent neutrophil infiltration and NETs formation (90). Huangqin Decoction (Scutellaria baicalensis-containing formula) suppresses experimental colitis-associated carcinogenesis by regulating PAD4-dependent NETs (42). Xianlian Jiedu Formula significantly inhibits CRC liver metastasis, and its mechanism may involve reducing neutrophil infiltration in the TME and suppressing NETs formation (91). The Astragalus membranaceus-Curcuma phaeocaulis herbal pair inhibits CRC liver metastasis by suppressing NETs generation and the NET-mediated VE-cadherin/β-catenin pathway (92). Future research may integrate network pharmacology to screen for potential TCM agents against NETs formation and validate them using CRC metastasis models, thereby opening new avenues for integrating Chinese and Western medicine to prevent and treat CRC metastasis.

### 5.5 Precision NET-targeting and combination strategies

Given the paradoxical nature of NETs in CRC, broad and indiscriminate inhibition of NETs formation risks eliminating the tumoricidal functions of NET-resident proteases (e.g., NE, CTSG) and compromising host anti-infective defense, as illustrated by the increased infection risk observed in animal models of systemic PAD4 or NE ablation. Accordingly, the field is shifting from global NETs suppression toward a more nuanced approach: (i) selectively dismantling the pro-tumorigenic arm of the nexus while preserving the anti-tumor arm; and (ii) integrating NET-directed interventions with standard-of-care therapies to maximize synergy.

Targeting NETs in combination with chemotherapy, radiotherapy, or immunotherapy holds strong synergistic potential. The PAD4 inhibitor GSK484 promotes cell death by inducing DNA double-strand breaks, suppresses NETs formation *in vivo*, and enhances the radiosensitivity of CRC (82). NETs are a major factor contributing to an “immune-desert” or “immune-excluded” TME. Targeting NETs may relieve their inhibition of T cells and increase intratumoral T-cell infiltration, converting “cold” tumors into “hot” ones and improving the efficacy of ICIs such as PD-1/PD-L1 inhibitors. Preclinical studies have already observed synergistic anti-tumor effects when DNase I is paired with anti-PD-1 therapy (63).

To minimize off-target NETs depletion and focus intervention within the TME, a range of precision delivery systems have been developed. A novel drug delivery system based on inulin-loaded oral chemotherapy microspheres improves cancer treatment by inhibiting NETs formation, modulating the gut microbiota, and reshaping the immune microenvironment (93). A photocurable hydrogel co-loaded with ^131^I and DNase I has been designed for intratumoral injection; upon ultraviolet-triggered *in situ* solidification, it achieves sustained local radioisotope retention while releasing DNase I to degrade radiation-induced NETs, thereby relieving immunosuppression and amplifying systemic anti-tumor immunity (94). Furthermore, solid lipid nanoparticles coated with orthogonal masked polyethylene glycol have been developed as co-delivery vehicles to target the valosin-containing protein inhibitor NMS-873 together with a bispecific PD-L1/cytotoxic T-lymphocyte-associated protein 4 aptamer to the TME. This strategy aims to overcome immunotherapy resistance in CRC by targeting valosin-containing protein, NETs, and dual immune checkpoints (95) Additionally, gene-therapy approaches such as adeno-associated virus-mediated hepatic DNase I expression can limit colorectal liver metastasis while preserving systemic anti-infective NETs responses (96).

## 6 Challenges and future perspectives

Despite considerable promise, several obstacles must be overcome before NET targeting can enter CRC clinical practice. First, targeting specificity and safety remain a core issue: how to selectively eliminate “pro-tumorigenic NETs” without compromising the normal anti-infective function of neutrophils is a central challenge in drug design, because NETs are important components of antimicrobial host defense, whereas excessive or tumor-associated NETs have been implicated in CRC progression and metastasis (51, 97, 98). Developing targeted drug delivery systems based on specific surface markers or tumor-homing properties of TANs may offer a pathway to improved safety, although validated TAN-specific markers and delivery strategies still require further optimization (99, 100). Second, biomarker development and clinical application are urgently needed to convert circulating NET markers, such as MPO-DNA and CitH3, or tissue NET density into clinical tools for prognostic stratification, response prediction, and dynamic monitoring (26, 31). Identifying CRC patients with high NET activity may be useful for interpreting NET-related biomarkers and evaluating NET-targeted therapies. However, NETs are highly heterogeneous structures, and their molecular composition is influenced by the signals that induce their formation (101). For example, NETs generated during infection, inflammation, or anticancer treatment may not have identical properties in primary tumors, metastatic lesions, or the circulation. Differences in neutrophil populations may further affect the DNA modifications, histone patterns, and granule proteins associated with NETs. These variations may partly account for the distinct biological effects of NETs, including metastasis, thrombosis, and immune regulation (37, 54). Proteomic studies and spatial imaging approaches may help clarify whether different NET populations exist and whether these populations respond differently to therapeutic intervention. Combination strategies also require careful evaluation. Rather than simply combining NET inhibitors with existing therapies, future studies should determine which treatment settings are most suitable for NET targeting, including drug sequence, administration time, and interaction with chemotherapy, radiotherapy, targeted therapy, or immunotherapy (48, 61). Notably, chemotherapy-induced NETs may either contribute to therapy resistance in some cancer contexts or mediate anti-tumor activity in defined CRC models, underscoring the need to define treatment context, timing, and sequence rather than uniformly blocking NETs. Fifth, focusing on perioperative management is critical. The perioperative period represents a golden window for NET intervention (35). Evaluating whether short-term preoperative or postoperative use of DNase I or PAD4 inhibitors can safely and effectively reduce postoperative metastasis and recurrence rates in CRC holds high translational value, because postoperative NET formation has been associated with poorer disease-free survival after liver resection for metastatic CRC, and NET degradation or PAD4 inhibition attenuated surgery-associated metastatic progression in preclinical models (65). Finally, the challenge is resolving the paradoxical nature of NETs in CRC. Recent authoritative reviews have likewise highlighted the importance of distinguishing tumor-promoting from tumor-restraining NET programs and have underscored neutrophil diversity and functional plasticity as key determinants of disease outcome (102). Therefore, future research should prioritize integrated multi-omics analyses, single-cell and spatial profiling of neutrophil subsets, and prospective clinical studies to identify patients most likely to benefit from context-aware, rather than broad-spectrum, NET-targeting therapies. Ultimately, precision modulation of pathogenic NET functions while preserving their physiological host-defense activities may represent the most promising direction for clinical translation.

## 7 Conclusion

NETs are involved in several stages of CRC, including colitis-associated tumor initiation, primary tumor growth, metastatic spread, treatment response, and CRC-related complications. In CRC models, NET production can be triggered by tumor-derived inflammatory mediators, stromal signals, microbial dysbiosis, oncogenic pathways, and ROS-dependent mechanisms. These findings connect neutrophil activation with inflammation, immune regulation, coagulation, and tumor progression.

The effects of NETs in CRC are not uniform. Most studies associate NETs with immune evasion, formation of the metastatic niche, and resistance to treatment. However, several experimental studies have described protective or anti-tumor effects of NETs under particular biological settings. Such divergent outcomes may be influenced by differences in tumor genetic background, neutrophil states, immune context, NET-inducing stimuli, and therapeutic interventions. These differences should be considered when interpreting NET-related studies, since broad inhibition of NET formation may not only affect pathological NET activity but also interfere with physiological neutrophil functions.

In this review, we discussed the mechanisms of NET formation during CRC development and summarized their reported roles in different disease settings. We also examined current NET-related biomarkers, detection strategies, and therapeutic approaches. Several issues still need to be clarified before NET-targeted strategies reach the clinic. These include establishing more consistent methods for NET detection, determining which patients exhibit NET-dependent tumor characteristics, and defining the appropriate context for NET inhibition. In particular, future work should distinguish between systemic suppression of NET formation and more selective approaches targeting specific NET-associated pathways or tissues. Resolving these questions will help determine how NET-directed therapies can be applied while preserving the protective functions of neutrophils.
